# *Aurantii Fructus Immaturus* enhances natural killer cytolytic activity and anticancer efficacy *in vitro* and *in vivo*

**DOI:** 10.3389/fmed.2022.973681

**Published:** 2022-08-18

**Authors:** Arum Park, Yunjeong Yang, Yunhee Lee, Haiyoung Jung, Tae-Don Kim, Ji-Yoon Noh, Seungjin Lee, Suk Ran Yoon

**Affiliations:** ^1^Immunotherapy Research Center, Korea Research Institute of Bioscience and Biotechnology, Daejeon, South Korea; ^2^Department of Pharmacology, College of Pharmacy, Chungnam University, Daejeon, South Korea

**Keywords:** *Aurantii Fructus Immaturus*, natural killer cells, cytotoxic activity, ERK signaling pathway, cancer immunotherapy

## Abstract

*Aurantii Fructus Immaturus* (AFI), extensively used in traditional herbal medicine, is known to have diverse physiological effects against various diseases, including obesity, diabetes, and cardiovascular disease. However, the effects of AFI on the immune system, especially natural killer (NK) cells, remain largely unknown. We aimed to investigate the effect of AFI on NK cell activity *in vitro* and *in vivo* and to elucidate the underlying mechanisms. Further, we verified the anticancer efficacy of AFI in a mouse lung metastasis model, underscoring the therapeutic potential of AFI in cancer therapy. Our results revealed that AFI significantly enhanced the cytolytic activity of NK cells in a dose-dependent manner, accompanied by an increase in the expression of NK cell-activating receptors, especially NKp30 and NKp46. AFI treatment also increased the expression of cytolytic granules, including granzyme B and perforin. Furthermore, the expression of CD107a, a degranulation marker, was increased upon treatment with AFI. A signaling study using western blot analysis demonstrated that the phosphorylation of extracellular signal-regulated kinase (ERK) was involved in increasing the NK cell activity following AFI treatment. In the *in vivo* study performed in mice, oral administration of AFI markedly enhanced the cytotoxic activity of spleen mononuclear cells against YAC-1 cells, which was accompanied by NKp46 upregulation. In addition, we confirmed that cancer metastasis was inhibited in a mouse cancer metastasis model, established using the mouse melanoma B16F10 cell line, by the administration of AFI *in vivo.* Collectively, these results indicate that AFI enhances NK cell-mediated cytotoxicity *in vitro* and *in vivo via* activation of the ERK signaling pathway and suggest that AFI could be a potential supplement for cancer immunotherapy.

## Introduction

Natural killer (NK) cells are white blood cells which form a part of the innate immune system and play a vital role in the first line of defense against cancer or virus-infected cells (1–3). Activated NK cells secrete cytolytic granules such as perforin (a membrane pore-forming protein) and granzyme B in a process known as degranulation (4, 5). During NK cell degranulation, lysosomal-associated membrane protein 1 (LAMP-1 or CD107a) is transiently expressed on the surface of NK cells. NK cell degranulation is often used as an indirect marker of NK cell activity (6). Perforin released in the target cells generates a pore, allowing granzyme to enter the cell and activate apoptosis of target cells *via* a caspase-dependent pathway (7–9). The functional activity of NK cells is controlled by activating and inhibitory receptors, including natural killer group 2D (NKG2D) and natural cytotoxicity receptors (NCRs) (NKp30, NKp46, and NKp44) (10–12). The ligation of activating receptors leads to the activation of intracellular signaling, resulting in the convergence of cytolytic granules to the microtubule-organizing center (MTOC) and its subsequent polarization with microtubules for its delivery to the synapse of target cells (13). NK cells with these characteristics play a crucial role in the immune response to tumors or bacterial and viral infections of the innate immune system (14). In mice, NK cells play a similar role; however, the types of receptors they express are somewhat different from those of human NK cells. In addition, the differentiation of NK cells in mice is divided into four subsets according to the expression of CD27 and CD11b. The roles and functions of NK cells in each subset are different; this subset classification is also used in humans (15, 16).

Melanoma is the most aggressive skin cancer of melanocytic origin and can metastasize to any organ, including the lungs, liver, bones, and brain (17). Metastatic melanoma is one of the most intractable cancers because of its ability to metastasize early and its resistance to conventional treatments. NK cells can recognize and destroy melanoma cell lines, which has also been demonstrated in murine models (18). NK cell-mediated changes (e.g., downregulation of active receptors or NK cell depletion) have been observed in patients with melanoma, which supports the development of escape mechanisms to avoid NK cell-mediated destruction (19). Therefore, the recognition and dissolution of melanoma cells by NK cells and a potential therapeutic strategy for melanoma based on NK cells are expected.

Many studies on anticancer drugs based on NK cells are actively underway and have focused on increasing the anticancer immune response by activating NK cells (20). Although several cytokines [such as interleukin (IL)-21, IL-15, and IL-2] are well known as NK cell activators (21–23), IL-2 is majorly used in clinical trials to increase the anti-tumor potential of NK cells. The use of high doses of IL-2 induces toxicity, which offsets its efficacy (24). To overcome this limitation, researchers have focused on developing natural compounds that enhance the activity of NK cells with few side effects (25, 26).

*Aurantii Fructus Immaturus* (AFI), harvested from May to June, is the dried immature fruit of bitter orange [*Citrus* × *aurantium* L. (Rutaceae)], summer orange (*Citrus natsudaidai* Hayata), and their cultivars (27, 28). It is widely used in traditional herbal formulas owing to its pharmacological activity. AFI has been commonly utilized for weight loss since it regulates adipogenesis *via* AMPK activation (29). It is also used as an antioxidant (30, 31), pesticide (32) and in treating cardiovascular diseases. AFI also has a therapeutic effect against rotavirus, which is a major cause of acute diarrhea (33). Additionally, AFI has been reported to have anticancer activities against human breast cancer cells (MCF-7) and lung cancer cells (HCC827) by stimulating the production of tumor necrosis factor-alpha (TNF-α), interleukin-6 (IL-6), and interleukin-1 beta (IL-1β) (34). However, the direct effects of AFI on the immune system, particularly NK cells, remain largely unknown.

Here, we demonstrated the potential of AFI as an anticancer drug candidate, which may act *via* the enhancement of NK cell activity. Our results showed that treatment with AFI significantly enhanced NK cell-mediated cytotoxicity in human cord blood mononuclear cells and mouse splenocytes through the induction of NK-activating receptors, especially NKp30 and NKp46, resulting in increased granzyme B expression. Moreover, we investigated the effect of the AFI extract on the inhibition of melanoma metastasis in a mouse melanoma model using B16F10 cells. Overall, our findings indicate the novel function of AFI as an NK cell booster and its potential for use in anti-tumor immunotherapy.

## Materials and methods

### Cell lines and culture

NK-92 cell lines were purchased from American Type Culture Collection (ATCC, Manassas, VA, United States). NK-92 cells were maintained in α-Minimum Essential Medium (MEM) containing 12.5% fetal bovine serum (FBS; Welgene, Gyeonsan, Korea), 12.5% horse serum (Gibco, Waltham, MA, United States), 0.2 mM inositol (Sigma-Aldrich, St. Louis, United States), 0.1 mM 2-mercaptoethanol (Sigma-Aldrich), 0.02 mM folic acid (Sigma-Aldrich) and 20 ng/μl IL-21 (PeproTech, Rocky Hill, United States).

K562 and YAC-1 cells, the human and mouse T cell lymphoma cells sensitive to NK cell-mediated lysis, were purchased from ATCC and cultured in RPMI containing 10% heat-inactivated FBS. The B16F10 murine melanoma cell line, which shows metastatic activity in the lungs of syngeneic C57BL/6 mice, was purchased from ATCC and cultured in DMEM containing 10% heat inactivated FBS. All tumor cell lines were cultured and maintained in a CO_2_ incubator at 37°C.

### Preparation of *Aurantii Fructus Immaturus*

Dried AFI powder was obtained from KOC biotech (Daejeon, Korea). This powder was obtained according to following protocol: 1 kg of mature fruit of *Citrus aurantium L* was extracted by reflux extraction with 5-fold ethanol for 3 h at 78 ± 2°C. The suspension was filtered with a 45 μm filter and concentrated using a rotary evaporator. The filtrate was freeze-dried which yielded 22.07% and stored at –20°C. AFI powder was dissolved in 1 × PBS and filtered using 45 um filter. This solution was used to treated NK cells.

### Natural killer cell proliferation and viability

The NK-92 cells were treated with various concentrations of AFI (ranging between 100 and 2,000 μg/mL) for 24 and 48 h, and the viability and cell proliferation of NK cells was examined using Cell Counting Kit-8 (CCK-8; Dojindo molecular technologies, Kumamoto, Japan) assay (Supplementary Figure 1A). An NK-92 cell suspension of 1 × 10^4^ cells was cultured in a 96-well microplate and treated with various concentrations of AFI for 24 and 48 h in a CO_2_ incubator at 37°C. Thereafter, 10 μl of CCK-8 was added to cells and then incubated for 3 h. Next, the optical density was detected at a wavelength of 450 nm using a microplate reader (Molecular Devices, San Jose, United States). To investigate the potential effects on NK cell activity, the concentration of AFI that did not affect the viability of NK cells was determined. In addition, NK cytotoxicity assay was performed against K562 target cells after treatment with AFI for 24 h (Supplementary Figure 1B).

### Natural killer cell isolation and culture

Human umbilical cord blood cells (UCBs) were collected from healthy women with full-term pregnancies, with the written informed consent of the mothers. The study was conducted in accordance with the Declaration of Helsinki, and all experimental procedures were approved by the Institutional Review Board (IRB) (IRB No. P-01-201610-31-002). Human NK cells were collected from the cord blood (CB) of healthy individuals. Briefly, the CD3 + cell fraction (T and NKT cells) of MNCs was eliminated by Rosette Sep (Stem Cell Technologies, Vancouver, Canada). The purity of CD3- cells was verified systematically by flow cytometry based on CD3 expression and then the cells were cultured in α-MEM supplemented with 10% FBS, 10 ng/mL human IL-15, 10 ng/mL IL-21, and10^–6^ M hydrocortisone (Stem Cell Technologies). All cytokines used for NK cell culture were purchased from PeproTech.

### Cell apoptosis

Induction of apoptosis was analyzed using Annexin V-FITC/PI apoptosis detection kit according to the manufacturer’s instructions. In brief, after 24 h treatment with 0, 50, and 100 μg/mL of AFI, the cells were double-stained with FITC-labeled annexin V and propidium iodide (PI) for each sample in the dark. Each sample was subjected to flow cytometry (Canto II, BD Bioscience) and analyzed using Flowjo software (TreeStar, Ashland, United States).

### Cytotoxicity assay

The cytotoxicity assay of NK cells was performed using calcein- acetoxymethyl (AM) release assay, as described previously (35). Before the assay, NK effector cells were cultured in IL-2 free medium for 24 h and the NK cells were pretreated either with or without ERK inhibitor (PD0325901) for 30 min, followed by AFI treatment for 24 h. Human leukemia cell line K562, and mouse leukemia cell line Yac-1 were used as target cells. The target cells were labeled with calcein-AM (Invitrogen, Carlsbad, United States) and co-cultured with the effector cells for 3 h. Then, the supernatant was collected and the amount of calcein released was measured using a multi-mode microplate reader (Molecular Devices, San Jose, United States). The percentage of specific lysis was calculated according to the formula: specific calcein-AM release = [(experimental release – minimum release)]/[(maximum release –minimum release)] × 100.

### Flow cytometry analysis

NK cells were stained with fluorescence labeled antibodies which were purchased from BD Bioscience (Franklin Lakes, United States) for further analysis. To analyze NK activating receptors on human cells, the cells were stained with BV-anti-CD56, APC-anti-NKG2D, APC-anti-NKp46, PE-anti-NKp44, and PE-anti-NKp30. For surface staining of mouse cells, FITC-anti-NK1.1, BV450-anti-B220, V500-anti-CD3, FITC-anti-CD4, PE-cy7-anti-CD8, PE-anti-CD27, PE-cy7-anti-CD11b, APC-anti-F4/80, FITC-anti-Gr1, BV421-anti-NKp46, and PE-anti-NKG2D were used. To examine the expression of cytolytic granules (granzyme B, perforin) and IFN- γ, NK cells were stained with APC-anti-CD56, fixed, and permeabilized for 30 min using Fixation/permeabilization buffer (BD bioscience, Franklin Lakes, United States). Next, the cells were labeled with BV450-anti-perforin, PE-anti-granzyme B, and PE-cy7-anti-IFN-γ for 20 min at 4°C in the dark. Finally, the cells were subjected to flow cytometry (Canto II, BD Bioscience) and analyzed using Flowjo software (TreeStar, Ashland, United States).

For the CD107a degranulation assay, NK-92 cells were treated either with or without AFI and incubated for 24 h. The NK-92 cells were co-cultured with K562 target cells (2.5:1 ratio) for 3 h. Then, anti-CD107a and anti-CD56 antibody were added for surface staining. The cells were harvested and washed with PBS containing 1% FBS. The fluorescence was analyzed using flow cytometry.

### Western blot analysis

Cell lysate was prepared in a RIPA buffer (Sigma-Aldrich) containing protease inhibitors. The amount of protein was measured using a BCA protein assay kit (Thermo Fisher Scientific, Waltham, United States). The lysates were resolved by 9% SDS-PAGE and transferred to a PVDF (Millipore, Bedford, United States) membrane. NK cells were cultured either by treatment with 50 and 100 μg/mL of AFI for 24 h, or pre-treated with ERK inhibitor (PD0325901, Sigma-Aldrich) for 30 min and then treated with 100 μg/mL of AFI for 24 h. ERK and JNK antibodies (Cell Signaling Technology, Danvers, United States) were used as primary antibodies and then incubated with appropriate HRP-conjugated secondary antibodies. The immune-reactive bands were visualized with Luminol/Enhancer solution and stable peroxide solution (Thermo Fisher Scientific, Waltham, United States). Densitometric quantification was performed using Image CSAnalyzer4 (ATTO Technology, United States). Beta-actin was used as an internal control.

### Natural killer cell activity after administration of *Aurantii Fructus Immaturus in vivo*

Six-week-old C57BL/6 male mice were obtained from Dooyeol Biotech (Seoul, Korea). The mice were acclimatized in a temperature- and humidity-controlled manner, with access to a standard diet and drinking water for 7 days.

Mice were randomly assigned to one of two groups: the control or AFI-treated group. The mice in the AFI treatment group were orally administered 200 mg/kg of AFI daily, and the control group received an equal volume of triple distilled water (3.D.W.) for 10 days. On day 10, the mice were sacrificed, and the spleens were extracted. The spleens were washed in RPMI 1640 medium containing an antibiotic-antimycotic mixture (Gibco) and homogenized using a syringe to collect the cells. The cells were treated with 1 × ACK buffer [0.15 NH_4_CL, 1.0 mM KHCO_3_, 0.1 mM EDTA (pH 7.4)] to remove the red blood cells. NK cell cytolytic activity against YAC-1 cells was evaluated in splenocytes using a calcein-AM release assay. NK cell-activating receptors and immune cell populations (B cells, NK cells, T cells, and macrophages) were examined using flow cytometry.

### Anti-tumor activity of *Aurantii Fructus Immaturus in vivo*

Mice were allowed to acclimatize for 1 week and were then randomly divided into one of three groups: the control, negative control (B16F10), or AFI treatment group. Mice in the negative control and AFI groups were injected intravenously (i.v.) with a single-cell suspension of B16F10 (5 × 10^5^ cells/100 mL) cells into the tail vein on day 0 and an equal volume of 1 × PBS in the control group on day 0. The control and B16F10 groups were given 3.D.W., and the AFI group were orally administered 200 mg/kg per day AFI. On day 15, the mice were sacrificed, and the lungs and spleens were collected. The numbers of tumor colonies on the lung surfaces were counted.

To evaluate the effect of AFI, immune cells were isolated from the lungs and spleens, and the cytotoxicity of NK cells, immune cell population, and IFN-γ expression levels were determined by flow cytometry.

### Statistical analysis

Data are expressed as mean ± standard error of the mean (S.E.M). Data were analyzed using Student’s *t*-test. Statistical significance was considered as a *p*-value of less than 0.05 (*), less than 0.01 (^**^), or less than 0.001 (^***^).

## Results

### *Aurantii Fructus Immaturus* enhanced the cytolytic activity of natural killer cells and natural killer receptor expressions

**FIGURE 1:**
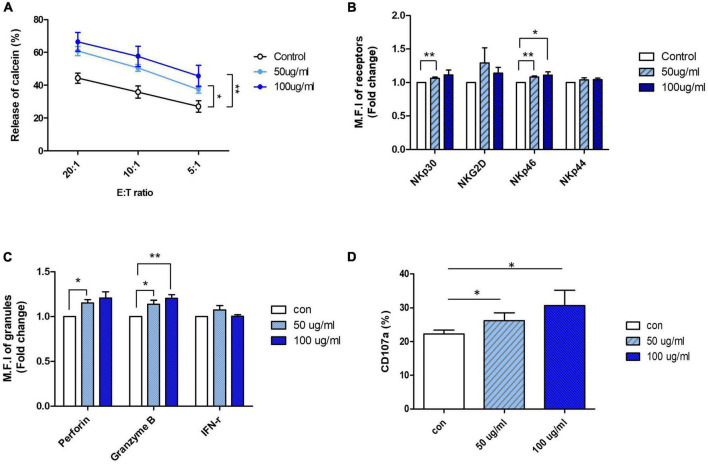
*Aurantii Fructus Immaturus* (AFI) enhance cytolytic activity of NK-92 cells. **(A)** NK-92 cells were cultured in the absence (empty) or presence of 50 μg/ml (sky blue) or 100 μg/ml (blue) of AFI for 24 h and cytotoxicity against K562 cells was examined. **(B)** NK cells were cultured in the absence (empty) or presence of 50 μg/ml (sky blue) or 100 μg/ml (blue) of AFI for 24 h. Expressions of NK receptors, including NKp30, NKG2D, NKp46, and NKp44 were detected by flow cytometry. Graphs represent the average values of fold changes in mean fluorescence intensity (MFI) of three independent experiments which were calculated based on the fact that MFI of each control is defined as 1. **(C)** Expressions of granules (Perforin, Granzyme B) and IFN-γ level in NK cells were analyzed by intracellular staining and flow cytometry. Graph represents the average fold changes in MFI of three independent experiments compared to control for each experiment. **(D)** Expression of CD107a on the surface of NK cells detected by flow cytometry. Graph indicates mean value of CD107a + expression percentage for three independent experiments. Bars represent the mean ± SEM of three independent experiments *p < 0.05 and ^**^p < 0.01.

Based on the results of the NK cell viability test and cytotoxicity assay, an appropriate range of AFI concentrations that did not show toxicity to the cells was determined: AFI concentrations of 50 and 100 μg/mL, which significantly increased NK cytotoxicity without undesirable toxicity, were used for further experiments. NK-92 cells were treated with 50 and 100 μg/mL AFI for 24 h, and cytotoxicity assays were performed using the human leukemia cell line K562 at effector: target (E:T) ratios of 5-, 10-, and 20-fold, respectively. As shown in Figure 1A, treatment with AFI significantly increased the NK cell cytotoxicity in a dose-dependent manner, and the NK activity was enhanced by 15–20% at a concentration of 100 μg/mL AFI, compared with the observations in the untreated control. It was confirmed by annexin V and propidium iodide (PI) staining that 50 and 100 μg/mL of AFI treatment did not affect the apoptosis of NK cells (Supplementary Figure 1C). This indicated that AFI did not affect the apoptosis of NK cells but rather enhanced their cytolytic activity against target cells. The cytotoxicity of NK cells is controlled by the balance between activating and inhibitory receptors expressed on their surfaces. In addition, the high expression of activating receptors triggers NK cell cytotoxicity mediated by cytolytic granules. Therefore, we investigated the effects of AFI on the expression of NK cell-activating receptors, such as NKG2D and NCRs. Flow cytometric analysis showed that the expression of the activating receptors was increased in NK cells treated with AFI, and the expression of NKp30 and NKp46 was significantly increased in a dose-dependent manner compared to that observed in the control group (Figure 1B).

Activated NK cells are known to induce the death of target cells by secreting IFN-γ and cytolytic granules such as perforin and granzyme B. Therefore, the expression of cytokines and granules in NK-92 cells treated with AFI was also investigated. The expression of perforin and granzyme B, measured using flow cytometry, was found to be significantly increased following AFI treatment, and the expression of IFN-γ was also slightly, although not significantly, increased (Figure 1C). Next, the expression of CD107a, a degranulation marker expressed on the surface of NK cells, was measured after AFI treatment to confirm whether the increased cytotoxicity of AFI is consistent with CD107a degranulation. After NK-92 cells were treated with AFI for 24 h, NK cells were incubated with K562 cells for 4 h, and the surface concentration of CD107a on CD56 cells was evaluated using flow cytometry. As shown in Figure 1D, AFI treatment increased the expression of CD107a in a dose-dependent manner. These data indicated that AFI induced the activation of NK cells by regulating the expression of NK cell-activating receptors, particularly NKp30 and NKp46, as well as the expression of cytolytic granules, so that AFI-treated NK cells killed target cells more effectively compared with untreated NK cells.

### *Aurantii Fructus Immaturus* enhanced the killing ability of natural killer cells through extracellular signal-regulated kinase activation

**FIGURE 2:**
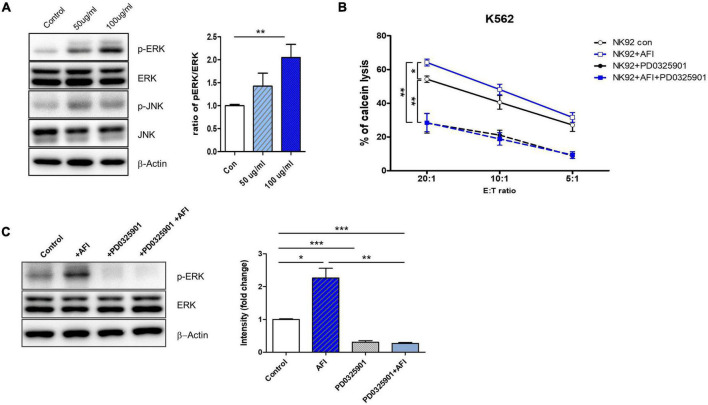
*Aurantii Fructus Immaturus* (AFI) enhances NK cell activity through ERK pathway. **(A)** Extracellular signal-regulated protein kinase (ERK) and c-Jun N-terminal kinases (JNK) analyzed by Western blotting after the treatment of AFI in NK cells. β-actin served as a loading control. Right graph represents relative intensity defined as the intensity of the ERK and p-ERK normalized to β-actin. NK cells were cultured for 24 h either alone, in the presence of AFI, in the presence of an inhibitor of ERK (PD0325901), or in the presence of AFI with the inhibitor of ERK. Thereafter **(B)** cytotoxicity and **(C)** ERK signal were investigated. Phosphorylation of ERK signals was examined by western blotting in NK cells under the indicated conditions. Right graph represents fold change values of intensity compared to control. Bars represent the mean ± SEM of three independent experiments *p < 0.05, **p < 0.01 and ***p < 0.001.

These activating receptors of NK cells have been reported to be upstream of the mitogen-activated protein kinase (MAPK) pathway. MAPK signaling pathways, including ERK and p38, are implicated in NK cell-mediated target cell killing, as well as in cytolytic granule and cytokine expression. The ERK pathway is involved in immune activation in NK cells. Eventually, ERK is phosphorylated by cytokines or activating receptors to induce NK cell activity. Activated NK cells produce cytokines and release granules (36–38). Thus, to investigate which molecule is regulated by AFI, NK-92 cells were treated with AFI for 24 h, and phosphorylation levels of ERK and p38 were examined by western blot analysis. As shown in Figure 2A, AFI significantly increased ERK kinase activity in a dose-dependent manner but had no significant effect on p38 phosphorylation (data not shown).

To verify whether AFI treatment plays a role in NK cell cytotoxicity through ERK activation, NK-92 cells were treated with AFI with or without the ERK inhibitor PD0325901 for 30 min before AFI treatment. After 100 μg/mL AFI treatment, ERK kinase activity was significantly increased compared to that in untreated cells. However, the killing abilities of NK-92 cells treated with PD0325901 only and NK-92 cells pretreated with PD0325901 before AFI treatment were significantly reduced (Figure 2B). The activation of ERK increased its phosphorylation, leading to enhanced NK cell cytotoxicity. As shown in Figure 2C, pretreatment with the ERK inhibitor PD0325901 (1 μM) attenuated the increase in ERK phosphorylation induced by AFI. These results indicated that AFI enhanced NK cell activity through the modulation of ERK activation.

### *Aurantii Fructus Immaturus* increased the cytolytic activity of primary natural killer cells derived from cord blood

**FIGURE 3:**
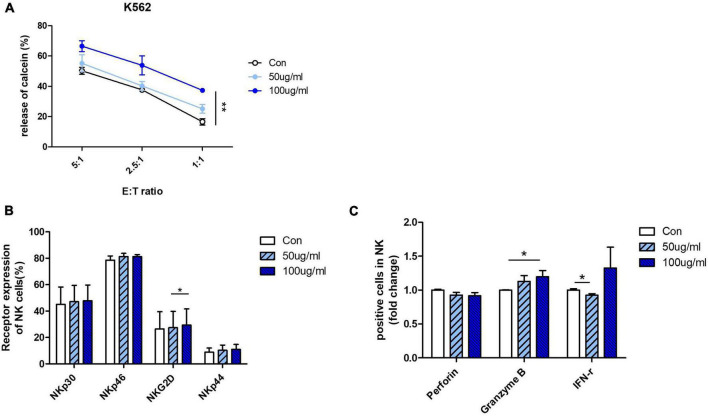
*Aurantii Fructus Immaturus* (AFI) enhances activity of primary NK cells from cord blood. **(A)** Matured primary NK cells were cultured in either the absence (empty) or presence of 50 μg/ml (sky blue) or 100 μg/ml (blue) of AFI for 24 h and cytotoxicity against K562 cells was examined. **(B)** Expressions of NK activating receptors analyzed by flow cytometry. Graphs represent mean value of expression percentage for three independent experiments. **(C)** Expressions of granules in NK cells were analyzed by intracellular staining and flow cytometry. Graphs represent fold change values of granule positive cells compared to control. Bars represent the mean ± SEM of three independent experiments *p < 0.05 and **p < 0.01.

Next, we confirmed the effect of AFI on cytolytic activity by using human primary NK cells instead of NK cell lines. NK cells differentiated from CD34 + cells from umbilical cord blood mononuclear cells were used. To confirm the efficacy of AFI in primary cells, mature NK cells expressing CD56 + CD3– were treated with AFI for 24 h. AFI treatment was as effective in primary NK cells as in NK-92 cells. NK cells treated with 100 μg/mL AFI showed a significant increase in cytolytic ability (Figure 3A). In addition, AFI treatment increased the expression of activating receptors in primary NK cells (Figure 3B). Granzyme B expression was also significantly increased in primary NK cells (Figure 3C). These results indicated that AFI affects NK cell activity in both NK cell lines as well as human-derived NK cells.

### *Aurantii Fructus Immaturus* affects the differentiation of natural killer cells from hematopoietic stem cells into functional natural killer cells

**FIGURE 4:**
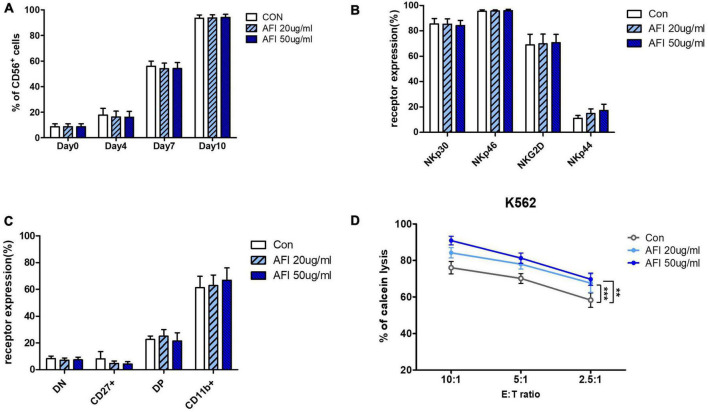
Effect of *Aurantii Fructus Immaturus* (AFI) on differentiation of NK Cells from HSCs. **(A)** Differentiation of NK cells was checked on days 0, 4, 7, and 10 after AFI treatment in NK cells. **(B)** Expression of NK activating receptors were analyzed by flow cytometry and graph was shown as mean values of expression percentage for three independent experiments. **(C)** Flow cytometry analysis of CD27 and CD11b expression was performed in gated CD56 + NK cells differentiated from CD34 + cells (hematopoietic stem cell marker) isolated from umbilical cord blood with and without AFI. The CD56 + NK cells were divided into four subsets based on the differential expression of CD27 and CD11b; double negative, CD27 + CD11b-, double positive, and CD27-CD11b+. **(D)** NK cells were differentiated in either the absence (empty) or presence of 50 μg/ml (sky blue) or 100 μg/ml (blue) of AFI for 10 days and cytotoxicity against K562 cells was examined. Bars represent the mean ± SEM of three independent experiments **p < 0.01 and ***p < 0.001.

We investigated whether AFI affects the differentiation of NK cells into hematopoietic stem cells (HSCs). HSCs were isolated from umbilical cord blood, differentiated into NK cell progenitors for 14 days, and then cultured in an mNK medium with or without AFI at 20 or 50 μg/mL. The levels of NK cell differentiation were determined in the CD56 + CD3– cell population using flow cytometry. As shown in Figure 4A, the AFI-treated groups had similar differentiation levels as the untreated group. Similarly, AFI treatment during NK cell differentiation did not significantly affect NK cell receptor expression (Figure 4B). Next, we identified four subsets of NK cells by CD27 and CD11b staining. It was confirmed that CD27–CD11b + cells, which had the highest killing ability among NK cells, occupied a greater proportion in the AFI-treated groups, but the difference was not significant (Figure 4C). In addition, no differences were observed in the expression levels of granules in mNK cells (Supplementary Figure 2). Nevertheless, the NK cells matured by treatment with AFI had a higher killing ability against K562 cells (Figure 4D). These results confirmed that AFI treatment during NK cell differentiation is helpful for differentiation into NK cells with high cytotoxicity.

### Administration of *Aurantii Fructus Immaturus* enhances natural killer cytolytic activity of splenocytes in mouse

**FIGURE 5:**
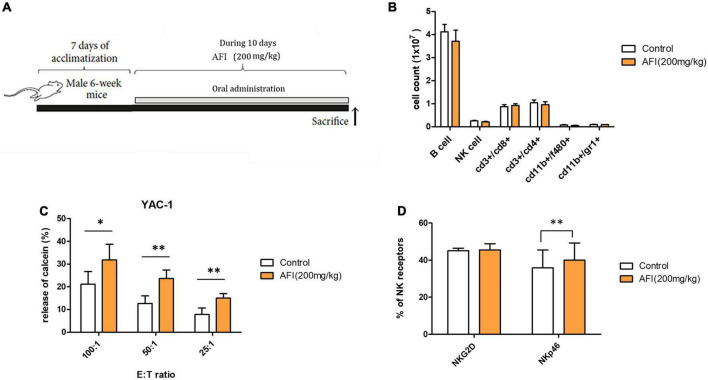
Administration of *Aurantii Fructus Immaturus* (AFI) enhances the cytolytic activity of natural killer (NK) cells *in vivo*. **(A)** Overall scheme to investigate the NK activity-inducing effect of AFI orally administered to mice. **(B)** Immune cells were analyzed in the mouse spleen after the end of the AFI dosing period by flow cytometry. Three independent experiments were conducted, with five mice per group. **(C)** Splenocytes from each group were isolated, and NK cytotoxicity was measured against YAC-1 cells according to the indicated E:T ratio. **(D)** After the AFI administration period, only NK cells were gated from splenocytes, and the expression of NKG2D and NKp46 was measured through flow cytometry. Bars represent the mean ± SEM of three independent experiments *p < 0.05 and ^**^p < 0.01.

To examine the *in vivo* effect of AFI, mice were orally administered AFI daily for 10 days (Figure 5A). For the experiment, 6-week-old mice were randomly divided into two groups: one group was administered AFI diluted in water at 200 μg/kg for 10 days, and the other was administered the same amount of water as the control group. After administration was completed, spleens were isolated from mice, and the distribution of immune cells in splenocytes was analyzed by flow cytometry. Oral administration of AFI was not related to an increase in NK cell population, represented as CD3–NK1.1 + in splenocytes. In addition, there was no significant difference in immune cell populations between the AFI-treated and untreated groups (Figure 5B). However, the NK cell activity of splenocytes against YAC-1 cells in the AFI treatment group was significantly increased compared to that in the untreated group (Figure 5C). In addition, the expression of NK activating receptors such as NKG2D and NKp46 was increased in the NK cells of mice administered AFI compared to those of the control group; in particular, increased NKp46 expression was significant (Figure 5D). Collectively, these results suggested that AFI had a significant effect on the functional activity of NK cells *in vivo* and *in vitro*.

### *Aurantii Fructus Immaturus* promotes anticancer immune activity of natural killer cells in the mouse metastasis model

**FIGURE 6:**
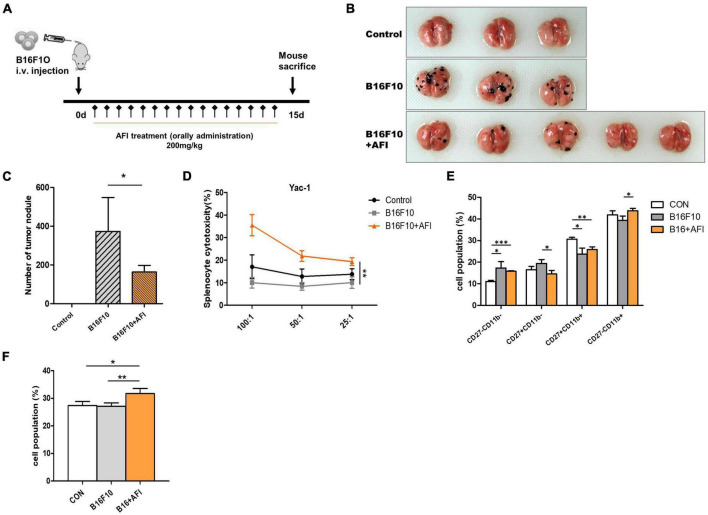
Administration of *Aurantii Fructus Immaturus* (AFI) inhibits cancer metastasis in a mouse melanoma model. **(A)** Overall scheme to investigate the antitumor-enhancing effect of orally administered AFI in mice. Lung metastasis of wild-type mice (control) and those that were orally administered AFI was analyzed 15 days after intravenous injection of B16F10 cells. **(B)** Representative images of the lung **(C)** and the number of melanoma nodules on the surface of the lung. **(D)** Measurement of cytotoxicity of three groups of splenocytes against YAC-1 cells. **(E)** Four subsets of natural killer (NK) cells using CD27 and CD11b markers **(F)** and IFN-γ positive NK cells were analyzed by flow cytometry in mouse splenocytes after B16F10 injection and AFI dosing periods *p < 0.05, **p < 0.01 and ***p < 0.001.

We conducted an experiment to confirm that AFI promotes anticancer efficacy, which constitutes one of the primary roles of NK cells, using a mouse metastasis model. To investigate whether AFI affects NK cell-mediated anti-tumor activity *in vivo*, B16F10 melanoma cells were injected intravenously into the tail vein, and AFI or water was administered for 14 days (39). Mice were divided into three groups; the “control” group was injected with 1 × PBS, the “B16F10” group was injected with 5 × 10^5^ B16F10 cells, and the “B16F10 + AFI” group was administered 200 mg/kg of AFI daily for 2 weeks after the same number of B16F10 cells were injected. The day after the end of the 2 weeks of administration, the mice were sacrificed, and the metastasis of cancer cells to the lungs was examined (Figure 6A). More tumor nodules were observed in the B16F10 group than in the control group, whereas fewer tumor nodules were observed in the group treated with AFI than in the B16F10 group (Figure 6B). Repeated experiments showed that AFI administration significantly reduced the metastasis of cancer cells (Figure 6C). The inhibition of metastasis may be due to the enhanced function of NK cells following AFI administration. To confirm this, splenocytes from each group were used as effector cells, and the cytotoxicity of NK cells was evaluated using YAC-1 cells, which are NK-specific target cells (40). The NK cell activity of splenocytes in the B16F10 group was decreased compared to that observed in the control group. However, the NK activity of splenocytes in the B16F10 + AFl group was significantly increased compared to that observed in the B16F10 group (Figure 6D). By analyzing NK cell subsets according to the expression of the CD27 and CD11B markers of splenocytes in each group, it was found that the CD27–CD11B + NK subset, which exhibits substantial cytotoxicity, decreased in the B16F10 group and increased in the AFI-treated group (Figure 6E). A similar distribution of NK subsets was observed in NK cells obtained from the lungs (Supplementary Figure 3). The proportion of IFN-γ positive cells in the spleen NK cells of each group also increased after AFI treatment *in vivo* (Figure 6F). These data suggested that the oral administration of AFI promotes the anticancer efficacy of NK cells *in vivo* through the activation of NK cells.

## Discussion

Dried AFI from the mature fruit of *C. aurantium* regulates various physiological functions, including those with antiviral, anti-obesity, and antioxidant effects. In particular, *Citrus* spp., which are abundant in vitamin C, reportedly play a more significant role in preventing cancer than any other food (41, 42). In this study, we demonstrated that AFI significantly enhances NK cell activity by upregulating NK cell-activating receptors *via* the ERK pathway. This enhancement of NK cell activity by AFI was confirmed by the observation of its anticancer efficacy in an *in vivo* mouse cancer metastasis model.

As shown in Supplementary Figure 1A, a higher AFI concentration caused lower cell viability. Concerning the effect of AFI concentration on NK cell activity, NK cell activity increased in proportion to the AFI concentration up to 100 μg/ml, but there was no significant change in NK cell activity at a concentration of 200 μg/ml or more (Supplementary Figure 1B). Therefore, the experiments were conducted at a concentration of 100 μg/ml or less. Treatment of NK cells with AFI did not significantly affect NK cell proliferation or differentiation. In contrast, it markedly increased NK cell cytotoxicity. NK cell cytotoxic activity appears to result from the activation of major NK cell-activating receptors such as NKG2D and NCRs, which trigger lysis of the target. NK cells with a high expression of NCRs have higher cytotoxicity than NK cells with a lower expression of NCRs (43–45). Therefore, the expression levels of NCRs were used to measure the activity of NK cells. In this study, we showed that AFI increased the expression of activating receptors on NK cells, particularly NKp30 and NKp46. Furthermore, NKp46 expression in NK cells was significantly increased following AFI treatment in a mouse model. Activated NK cells synthesize cell granules, such as perforin and granzyme B, and secrete them out of the cell to lyse the target cells (46, 47). Treatment with AFI increased the gene expression of perforin and significantly increased the expression of granzyme B. NK cell cytotoxicity was confirmed not only in the NK-92 cell line but also in primary NK cells (Figure 4).

After obtaining only CD34 + cells isolated from cord blood, we conducted an experiment using primary NK cells differentiated in an NK maturation medium (48). This experiment confirmed that AFI affects both simple cell lines and human-derived NK cells. AFI did not show the same effect in increasing NK-activating receptor expression in both cell lines and human-derived NK cells. However, AFI increased the expression of granzyme B and increased the killing ability of NK cells.

NK cells play a vital role in activating acquired immunity by secreting various cytokines and chemokines, such as interferon (IFN)-γ, tumor necrosis factor (TNF)-α, macrophage inflammatory protein-1 (MIP-1) α, and MIP-1β (49–51). In particular, activated NK cells increase IFN-γ secretion, activating dendritic cells, thereby increasing innate immunity against pathogens. No significant effect was observed on the IFN-γ expression in NK-92 cells treated with AFI (Figure 1C). However, IFN-γ production in the mouse anticancer model increased after AFI was administered (Figure 6F). Owing to the dose-dependent difference in cytokine production, it is also required to verify the optimal concentration based on cytokine production.

According to previous studies, the expression of the NK activating receptor is stimulated *via* the MAPK pathways. The MAPK pathway is a crucial signal in NK cell cytotoxicity through perforin and granzyme B movement toward target cells (52, 53). ERK activation is achieved through various pathways, and representative examples include phosphatidylinositol 3-kinase (PI3K)–ras-related C3 botulinum toxin substrate 1 (Rac1)–p21-activated kinase (PAK)–MAPK kinase (MEK) and Janus kinase (JAK)–signal transducer and activator of transcription (STAT), which activate NK cells, leading to increased cytotoxicity (13, 35, 54). Accordingly, we showed that the ERK pathway is specifically activated by treatment with AFI. In addition, our results showed that the ERK inhibition of NK-92 cells effectively suppressed AFI-induced cytotoxicity, suggesting that the ERK pathway is implicated in the enhanced NK cell activity induced by AFI. The blockage of PI3K results in the inhibition of the activation of PAK1, MEK, and ERK and the movement of cytolytic granules to the target cells, resulting in decreased NK cytotoxicity. One possible explanation is that the NCR signal is known to pass through the PI3K–Rac–ERK axis, and AFI may increase the expression of PI3K and Rac molecules because it upregulates the expression of NKp30 and NKp46.

In this study, we demonstrated the NK cell activation effect of AFI in cell lines, primary cells, and in mice; however, the signaling pathway and downstream mechanisms are still not understood clearly. Therefore, further research is required to identify the role of the exact molecules upregulated by AFI in signaling pathways. Overall, our findings indicated that AFI markedly increased the K562 lysis of NK cells and elevated granzyme B expression. Nonetheless, AFI treatment could increase NK activating receptors, such as NKp46 and NKp30. Furthermore, the oral intake of AFI enhances NK cell cytotoxicity and NKp46 expression. Collectively, we propose that AFI is an immunostimulator that enhances NK cell activity for cancer treatment.

## Data availability statement

The original contributions presented in this study are included in the article/Supplementary material, further inquiries can be directed to the corresponding author.

## Ethics statement

The studies involving human participants were reviewed and approved by Dong-A College of Medicine. The patients/participants provided their written informed consent to participate in this study. This animal study was reviewed and approved by Korea Research Institute of Bioscience and Biotechnology.

## Author contributions

AP and YY planned the experiments, analyzed the data, and drafted the manuscript. AP, YY, and YL performed experiments. HJ, T-DK, J-YN, and SL provided useful comments and suggestions. SY designed and supervised all experiments and wrote the manuscript. All authors have read and approved the final manuscript prior to submission.

## References

[1] Farag SS Caligiuri MA. Human natural killer cell development and biology. *Blood Rev.* (2006) 20:123–37. 10.1016/j.blre.2005.10.001 16364519

[2] Somanchi SS Senyukov VV Denman CJ Lee DA. Expansion, purification, and functional assessment of human peripheral blood NK cells. *J Vis Exp.* (2011) 48:2540. 10.3791/2540 21339714 PMC3180743

[3] Vivier E Tomasello E Baratin M Walzer T Ugolini S. Functions of natural killer cells. *Nat Immunol.* (2008) 9:503–10. 10.1038/ni1582 18425107

[4] Fehniger TA Cai SF Cao X Bredemeyer AJ Presti RM French AR Acquisition of murine NK cell cytotoxicity requires the translation of a pre-existing pool of granzyme B and perforin mRNAs. *Immunity.* (2007) 26:798–811. 10.1016/j.immuni.2007.04.010 17540585

[5] Ogbomo H Mody CH. Granule-dependent natural killer cell cytotoxicity to fungal pathogens. *Front Immunol.* (2016) 7:692. 10.3389/fimmu.2016.00692 28123389 PMC5225108

[6] Alter G Malenfant JM Altfeld M. CD107a as a functional marker for the identification of natural killer cell activity. *J Immunol Methods.* (2004) 294:15–22. 10.1016/j.jim.2004.08.008 15604012

[7] Martinez-Lostao L Anel A Pardo J. How do cytotoxic lymphocytes kill cancer cells? *Clin Cancer Res.* (2015) 21:5047–56. 10.1158/1078-0432.CCR-15-0685 26567364

[8] Pardo J Balkow S Anel A Simon MM. Granzymes are essential for natural killer cell-mediated and perf-facilitated tumor control. *Eur J Immunol.* (2002) 32:2881–7. 10.1002/1521-4141(2002010)32:10<2881::AID-IMMU2881>3.0.CO;2-K 12355441

[9] Topham NJ Hewitt EW. Natural killer cell cytotoxicity: How do they pull the trigger? *Immunology.* (2009) 128:7–15. 10.1111/j.1365-2567.2009.03123.x 19689731 PMC2747134

[10] Campbell KS Yusa S Kikuchi-Maki A Catina TL. NKp44 triggers NK cell activation through DAP12 association that is not influenced by a putative cytoplasmic inhibitory sequence. *J Immunol.* (2004) 172:899–906. 10.4049/jimmunol.172.2.899 14707061

[11] Elboim M Gazit R Gur C Ghadially H Betser-Cohen G Mandelboim O. Tumor immunoediting by NKp46. *J Immunol.* (2010) 184:5637–44. 10.4049/jimmunol.0901644 20404273

[12] Li Y Wang Q Mariuzza RA. Structure of the human activating natural cytotoxicity receptor NKp30 bound to its tumor cell ligand B7-H6. *J Exp Med.* (2011) 208:703–14. 10.1084/jem.20102548 21422170 PMC3135353

[13] Li C Ge B Nicotra M Stern JN Kopcow HD Chen X JNK MAP kinase activation is required for MTOC and granule polarization in NKG2D-mediated NK cell cytotoxicity. *Proc Natl Acad Sci USA.* (2008) 105:3017–22. 10.1073/pnas.0712310105 18287025 PMC2268577

[14] Yokoyama WM Kim S French AR. The dynamic life of natural killer cells. *Annu Rev Immunol.* (2004) 22:405–29. 10.1146/annurev.immunol.22.012703.104711 15032583

[15] Fu B Wang F Sun R Ling B Tian Z Wei H. CD11b and CD27 reflect distinct population and functional specialization in human natural killer cells. *Immunology.* (2011) 133:350–9. 10.1111/j.1365-2567.2011.03446.x 21506999 PMC3112344

[16] Chiossone L Chaix J Fuseri N Roth C Vivier E Walzer T. Maturation of mouse NK cells is a 4-stage developmental program. *Blood.* (2009) 113:5488–96. 10.1182/blood-2008-10-187179 19234143

[17] Redmer T. Deciphering mechanisms of brain metastasis in melanoma – The gist of the matter. *Mol Cancer.* (2018) 17:106. 10.1186/s12943-018-0854-5 30053879 PMC6064184

[18] Kim WS Kim MJ Kim DO Byun JE Huy H. Suppressor of cytokine signaling 2 negatively regulates NK cell differentiation by inhibiting JAK2 activity. *Sci Rep.* (2017) 7:46153. 10.1038/srep46153 28383049 PMC5382670

[19] Lee H Da Silva IP Palendira U Scolyer RA Long GV Wilmott JS. Targeting NK cells to enhance melanoma response to immunotherapies. *Cancers (Basel).* (2021) 13:1363. 10.3390/cancers13061363 33802954 PMC8002669

[20] Carotta S. Targeting NK cells for anticancer immunotherapy: Clinical and preclinical approaches. *Front Immunol.* (2016) 7:152. 10.3389/fimmu.2016.00152 PMC4838611 27148271

[21] Sim GC Radvanyi L. The IL-2 cytokine family in cancer immunotherapy. *Cytokine Growth Factor Rev.* (2014) 25:377–90. 10.1016/j.cytogfr.2014.07.018 25200249

[22] Wu Y Tian Z Wei H. Developmental and functional control of natural killer cells by cytokines. *Front Immunol.* (2017) 8:930. 10.3389/fimmu.2017.00930 28824650 PMC5543290

[23] Huntington ND. The unconventional expression of IL-15 and its role in NK cell homeostasis. *Immunol Cell Biol.* (2014) 92:210–3. 10.1038/icb.2014.1 24492800

[24] Childs RW Carlsten M. Therapeutic approaches to enhance natural killer cell cytotoxicity against cancer: The force awakens. *Nat Rev Drug Discov.* (2015) 14:487–98. 10.1038/nrd4506 26000725

[25] Grudzien M Rapak A. Effect of natural compounds on NK cell activation. *J Immunol Res.* (2018) 2018:4868417. 10.1155/2018/4868417 30671486 PMC6323526

[26] Cifaldi L Locatelli F Marasco E Moretta L Pistoia V. Boosting natural killer cell-based immunotherapy with anticancer drugs: A perspective. *Trends Mol Med.* (2017) 23:1156–75. 10.1016/j.molmed.2017.10.002 29133133

[27] Bai Y Zheng Y Pang W Peng W Wu H Yao H Identification and comparison of constituents of *Aurantii Fructus* and *Aurantii Fructus Immaturus* by UFLC-DAD-Triple TOF-MS/MS. *Molecules.* (2018) 23:803. 10.3390/molecules23040803 29601542 PMC6017871

[28] Wu J Huang G Li Y Li X. Flavonoids from *Aurantii Fructus Immaturus* and *Aurantii Fructus*: Promising phytomedicines for the treatment of liver diseases. *Chin Med.* (2020) 15:89. 10.1186/s13020-020-00371-5 32863858 PMC7449045

[29] Park J Kim HL Jung Y Ahn KS Kwak HJ Um JY. Bitter Orange (*Citrus aurantium* Linne) improves obesity by regulating adipogenesis and thermogenesis through AMPK activation. *Nutrients.* (2019) 11:1988. 10.3390/nu11091988 31443565 PMC6770725

[30] Sun Y Qiao L Shen Y Jiang P Chen J Ye X. Phytochemical profile and antioxidant activity of physiological drop of citrus fruits. *J Food Sci.* (2013) 78:C37–42. 10.1111/j.1750-3841.2012.03002.x 23301602

[31] Su Y Li L. Structural characterization and antioxidant activity of polysaccharide from four auriculariales. *Carbohydr Polym.* (2020) 229:115407. 10.1016/j.carbpol.2019.115407 31826485

[32] da Camara CAG Akhtar Y Isman MB Seffrin RC Born FS. Repellent activity of essential oils from two species of Citrus against *Tetranychus urticae* in the laboratory and greenhouse. *Crop Protect.* (2015) 74:110–5. 10.1016/j.cropro.2015.04.014

[33] Kim DH Song MJ Bae EA Han MJ. Inhibitory effect of herbal medicines on rotavirus infectivity. *Biol Pharm Bull.* (2000) 23:356–8. 10.1248/bpb.23.356 10726895

[34] Shen CY Yang L Jiang JG Zheng CY Zhu W. Immune enhancement effects and extraction optimization of polysaccharides from *Citrus aurantium* L. var. amara Engl. *Food Funct.* (2017) 8:796–807. 10.1039/c6fo01545j 28121002

[35] Park A Yang Y Lee Y Kim MS Park Y-J Jung H Indoleamine-2,3-dioxygenase in thyroid cancer cells suppresses natural killer cell function by inhibiting NKG2D and NKp46 expression via STAT signaling pathways. *J Clin Med.* (2019) 8:842. 10.3390/jcm8060842 31212870 PMC6617210

[36] Yu TK Caudell EG Smid C Grimm EA. IL-2 activation of NK cells: Involvement of MKK1/2/ERK but not p38 kinase pathway. *J Immunol.* (2000) 164:6244–51. 10.4049/jimmunol.164.12.6244 10843677

[37] Yang L Shen M Xu LJ Yang X Tsai Y Keng PC Enhancing NK cell-mediated cytotoxicity to cisplatin-resistant lung cancer cells via MEK/Erk signaling inhibition. *Sci Rep.* (2017) 7:7958. 10.1038/s41598-017-08483-z 28801607 PMC5554231

[38] Park A Lee Y Kim MS Kang YJ Park YJ Jung H Prostaglandin E2 secreted by thyroid cancer cells contributes to immune escape through the suppression of natural killer (NK) cell cytotoxicity and NK cell differentiation. *Front Immunol.* (2018) 9:1859. 10.3389/fimmu.2018.01859 30140269 PMC6094168

[39] Kodama T Takeda K Shimozato O Hayakawa Y Atsuta M Kobayashi K Perforin-dependent NK cell cytotoxicity is sufficient for anti-metastatic effect of IL-12. *Eur J Immunol.* (1999) 29:1390–6. 10.1002/(SICI)1521-4141(199904)29:04<1390::AID-IMMU1390>3.0.CO;2-C 10229107

[40] Wright SC Bonavida B. YAC-1 variant clones selected for resistance to natural killer cytotoxic factors are also resistant to natural killer cell-mediated cytotoxicity. *Proc Natl Acad Sci USA.* (1983) 80:1688–92. 10.1073/pnas.80.6.1688 6572932 PMC393668

[41] Kim K-S Yang HJ Choi E-K Shin MH Kim K-H Um JY The effects of complex herbal medicine composed of Cornus fructus, Dioscoreae rhizoma, Aurantii fructus, and Mori folium in obese type-2 diabetes mice model. *Orient Pharm Exp Med.* (2013) 13:69–75. 10.1007/s13596-013-0107-5

[42] Liu XY Fan ML Wang HY Yu BY Liu JH. Metabolic profile and underlying improved bio-activity of *Fructus Aurantii Immaturus* by human intestinal bacteria. *Food Funct.* (2017) 8:2193–201. 10.1039/c6fo01851c 28504280

[43] Joncker NT Fernandez NC Treiner E Vivier E Raulet DH. NK cell responsiveness is tuned commensurate with the number of inhibitory receptors for self-MHC class I: The rheostat model. *J Immunol.* (2009) 182:4572–80. 10.4049/jimmunol.0803900 19342631 PMC2938179

[44] Kumar S. Natural killer cell cytotoxicity and its regulation by inhibitory receptors. *Immunology.* (2018) 154:383–93. 10.1111/imm.12921 29512837 PMC6002213

[45] Pegram HJ Andrews DM Smyth MJ Darcy PK Kershaw MH. Activating and inhibitory receptors of natural killer cells. *Immunol Cell Biol.* (2011) 89:216–24. 10.1038/icb.2010.78 20567250

[46] Krensky AM Clayberger C. Granulysin: A novel host defense molecule. *Am J Transplant.* (2005) 5:1789–92. 10.1111/j.1600-6143.2005.00970.x 15996224

[47] de Saint Basile G Menasche G Fischer A. Molecular mechanisms of biogenesis and exocytosis of cytotoxic granules. *Nat Rev Immunol.* (2010) 10:568–79. 10.1038/nri2803 20634814

[48] Yoon SR Chung JW Choi I. Development of natural killer cells from hematopoietic stem cells. *Mol Cells.* (2007) 24:1–8. 17846493

[49] Bluman EM Bartynski KJ Avalos BR Caligiuri MA. Human natural killer cells produce abundant macrophage inflammatory protein-1 alpha in response to monocyte-derived cytokines. *J Clin Invest.* (1996) 97:2722–7. 10.1172/JCI118726 8675682 PMC507364

[50] Roda JM Parihar R Magro C Nuovo GJ Tridandapani S Carson WE III. Natural killer cells produce T cell-recruiting chemokines in response to antibody-coated tumor cells. *Cancer Res.* (2006) 66:517–26. 10.1158/0008-5472.CAN-05-2429 16397268

[51] Fauriat C Long EO Ljunggren HG Bryceson YT. Regulation of human NK-cell cytokine and chemokine production by target cell recognition. *Blood.* (2010) 115:2167–76. 10.1182/blood-2009-08-238469 19965656 PMC2844017

[52] Chini CC Boos MD Dick CJ Schoon RA Leibson PJ. Regulation of p38 mitogen-activated protein kinase during NK cell activation. *Eur J Immunol.* (2000) 30:2791–8. 11069059 10.1002/1521-4141(200010)30:10<2791::AID-IMMU2791>3.0.CO;2-D

[53] Vivier E Nunes JA Vely F. Natural killer cell signaling pathways. *Science.* (2004) 306:1517–9. 10.1126/science.1103478 15567854

[54] Gotthardt D Trifinopoulos J Sexl V Putz EM. JAK/STAT cytokine signaling at the crossroad of NK cell development and maturation. *Front Immunol.* (2019) 10:2590. 10.3389/fimmu.2019.02590 31781102 PMC6861185

