# Supplementary material for: Aurantii Fructus Immaturus enhances natural killer cytolytic activity and anticancer efficacy in vitro and in vivo
Source: Front Med (Lausanne). 2022 Aug 18;9:973681. doi: 10.3389/fmed.2022.973681 (PMC9433751; doi:10.3389/fmed.2022.973681)
Supplement: Supplementary file 1 [file Data_Sheet_1.DOCX]

Supplementary Figures

#


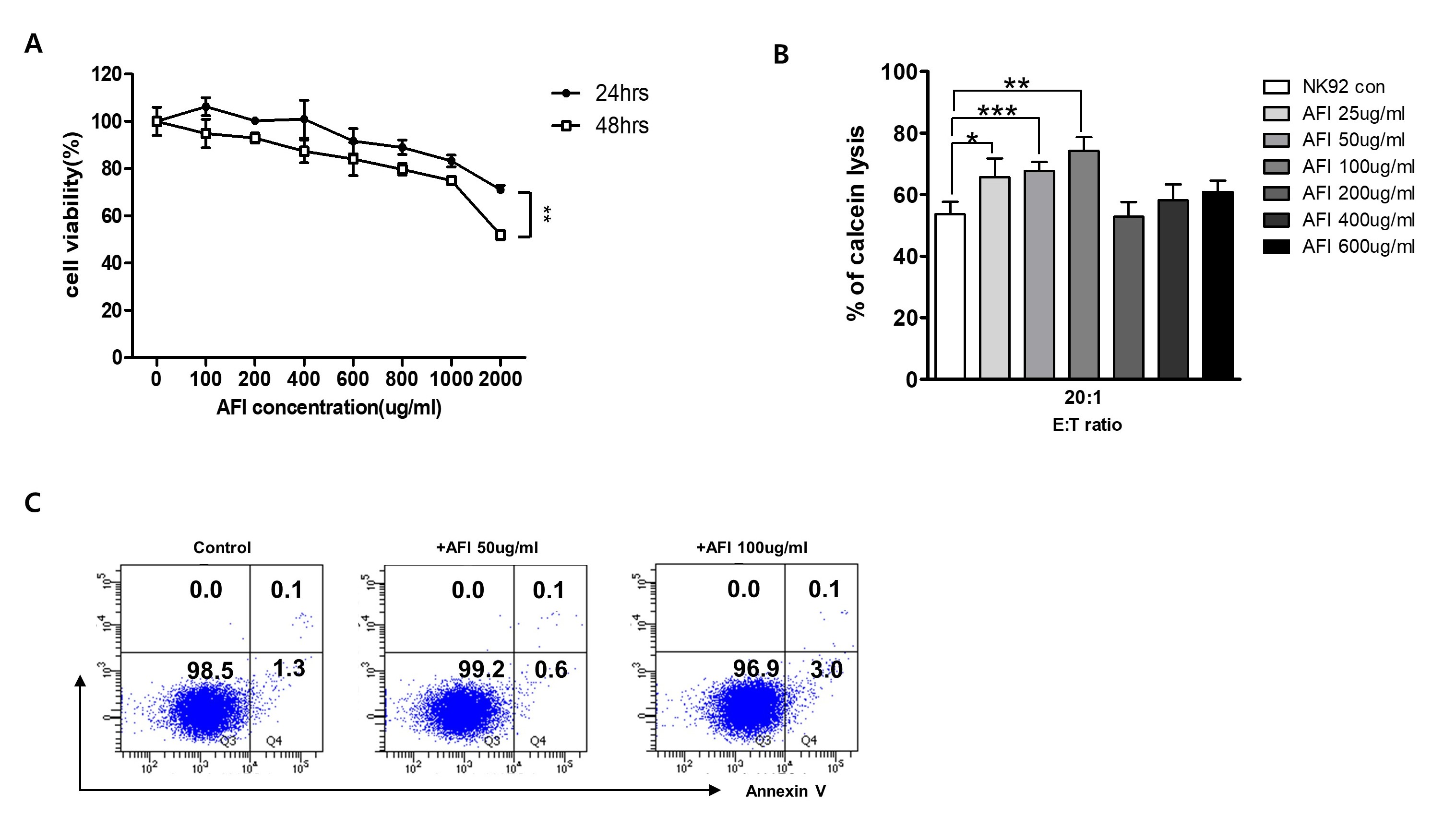


**Supplementary Figure 1.** Effect of AFI on toxicity and viability of NK-92 cells. (A) The toxicity and viability of NK-92 cells were examined at various concentrations of AFI treatment for 24 h and 48 h using cck-8 assay. (B) NK-92 cells were cultured in the absence (NK92 con) or presence of various concentrations of AFI for 24 h and cytotoxicity assay against K562 cells was performed. (C) Apoptosis of NK-92 cells was examined using annexin V / PI after incubation for 24 h in the absence or presence of 50 μg/ml or 100 μg/ml of AFI.


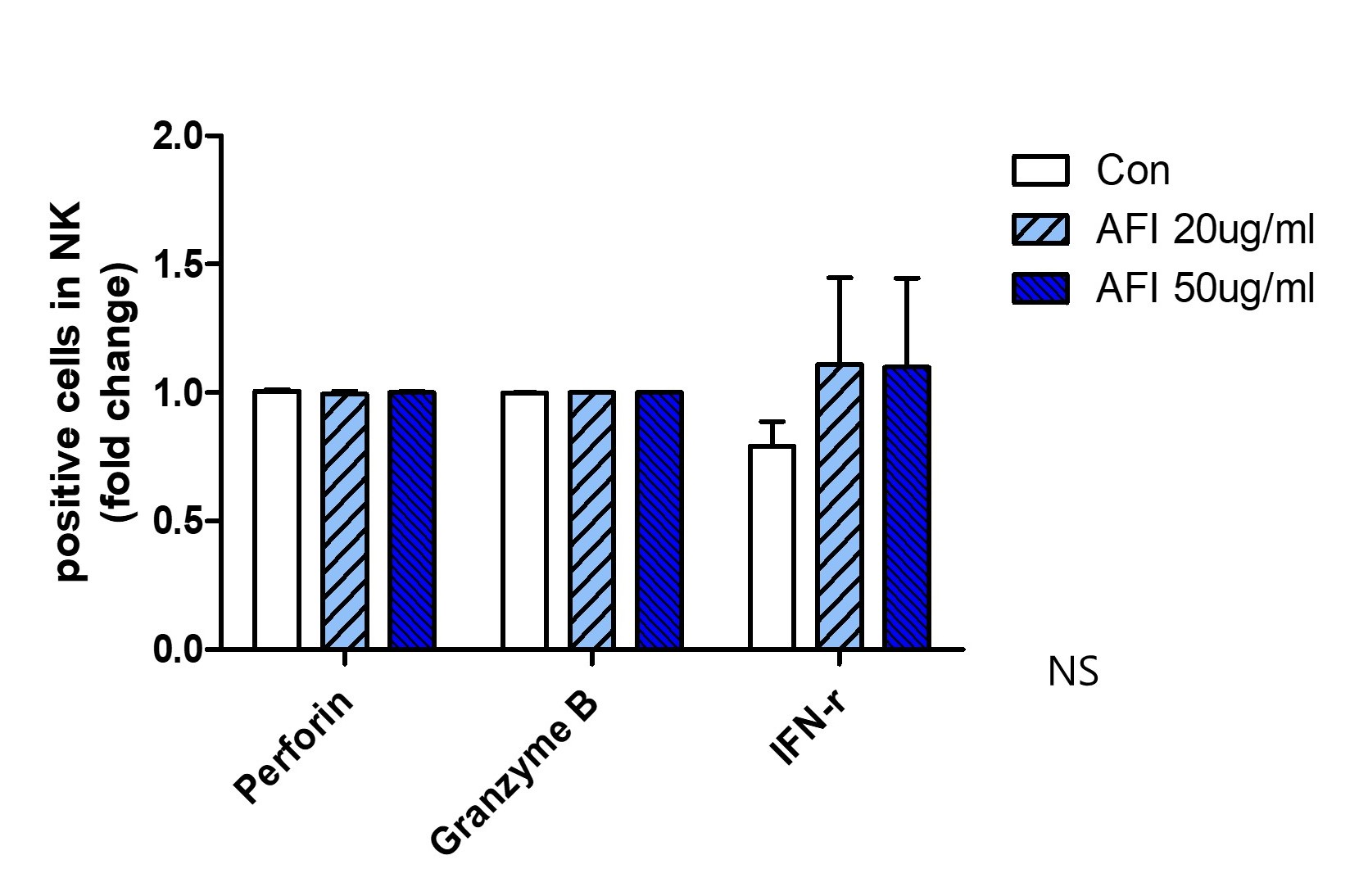


**Supplementary Figure 2.** The fold changes in expression of granules and IFN-γ of NK cells treated with AFI during NK differentiation.


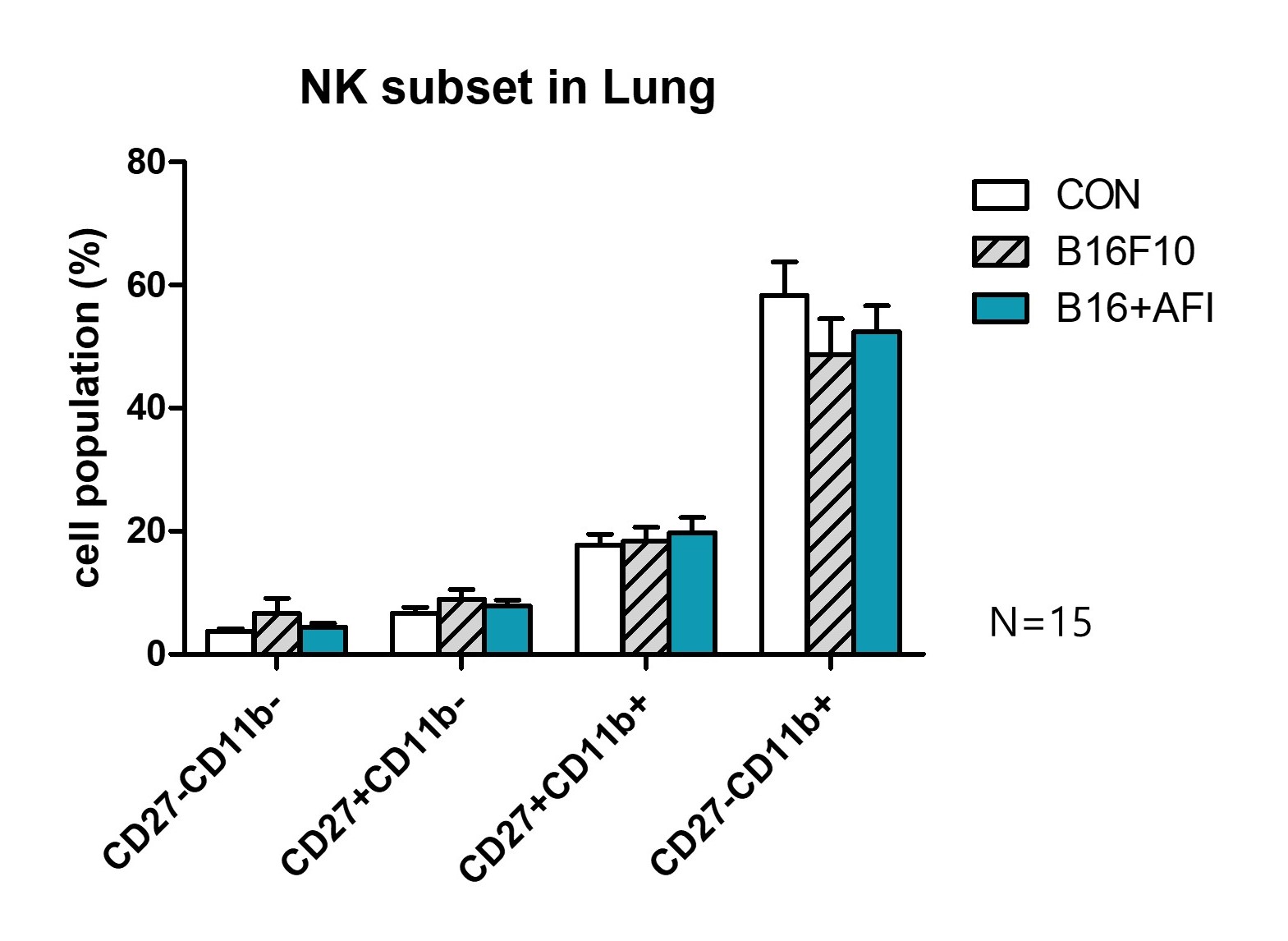


**Supplementary Figure 3.** Four subsets of natural killer (NK) cells were analyzed by flow cytometer based on the expression of CD27 and CD11b in lung NK cells.
